# Remodeling Cell Adhesion Releases Cardiac Potential of Human Pluripotent Stem Cells with Continuous Proliferation and Accelerated Maturation

**DOI:** 10.7150/ijbs.120853

**Published:** 2025-10-10

**Authors:** Weiwei Liu, Chuyu Liu, Qian Wang, Chengwu Li, Jiaxian Wang, Ning-Yi Shao, Guokai Chen

**Affiliations:** 1 Centre of Reproduction, Development and Aging, Faculty of Health Sciences, University of Macau, Macau SAR, China.; 2 Biological Imaging and Stem Cell Core Facility, Faculty of Health Sciences, University of Macau, Macau SAR, China.; 3 Department of Biomedical Sciences, Faculty of Health Sciences, University of Macau, Macau SAR, China.; 4 HELP Stem Cell Innovations Ltd. Co., Nanjing, Jiangsu, China.; 5 MoE Frontiers Science Center for Precision Oncology, University of Macau, Macau SAR, China.

## Abstract

Human pluripotent stem cells (hPSCs) can generate specific cell types for therapeutic applications. Since cell therapy often requires billions of cells for transplantation, it is essential to maximize differentiation efficiency to optimize both cell yield and quality. Cardiomyocytes are commonly induced in static culture with limited expandability. In this study, we explored the impact of cell adhesion remodeling on hPSC cell fate determination. We reveal that cell passaging at critical time points drives cardiac cell fate even without traditional cardiac inducers. Cardiac fate is specified while cells proliferate continuously. Cell adhesion remodeling leads to a 10-fold increase of cardiomyocyte yield with high purity in comparison to traditional static culture. AMPK activation and PI3K/AKT inhibition were observed following cell passaging. The impact of cell passaging can be mimicked by Src and FAK inhibition, suggesting critical roles of integrin signaling pathway in passaging-driven cardiac differentiation. Transcriptome analysis suggests that cell adhesion remodeling enhances the expression of critical cardiac genes associated with maturation. This study highlights that cell adhesion remodeling significantly impacts cell fate during *in vitro* differentiation. Our study provides an ideal method for high-yield, high-purity cardiomyocyte production, and offers a useful potential strategy for generating other cell types through directed differentiation.

## Introduction

Human pluripotent stem cells (hPSCs), including human embryonic stem cells (hESCs) and induced pluripotent stem cells (ihPSCs), can generate all cell types in our body. hPSCs are ideal starting materials to generate functional cells such as cardiomyocytes in large quantities for drug screening and cell therapies. The ability to produce high-quality functional cells with high yield is vital for translation of hPSC technology into clinical applications.

Monolayer culture is a common platform for lineage-specific differentiation, including cardiomyocytes and other cell types. hPSCs are usually induced with specific signaling modulators while cultured on extracellular matrices in confined vessels till the desired cell type appears in the end. On such static platforms, hPSCs usually reach confluence and stop proliferation in the first few days of differentiation, often with medium acidosis and contact inhibition due to high cell density 1. Such a highly stressful environment is commonly seen in various *in vitro* differentiation procedures, but its impact on cell fate determination is rarely discussed. It is interesting and important to understand whether interactions between hPSCs and the extracellular matrix have an impact on cell fate determination, and to explore the possibility of improving differentiation through modulation of this interaction at a critical time point. For example, in conventional cardiomyocyte generation protocols, hPSCs are first induced to mesendoderm progenitor cells by WNT pathway activation, and are then specified to cardiac fate by WNT inhibition 2-4. Without growth factor or chemical modulators, mesendoderm progenitors will spontaneously differentiate and generate a variety of cell types 5. It is unclear how the modulation of cell adhesion would affect cell fate determination from mesendoderm progenitors, which is the focus of this study.

During embryogenesis, cells constantly proliferate while various cell fates are specified from gastrulation to organogenesis. Meanwhile, frequent cell migration and rearrangement of cell adhesion are observed in the process 6, 7. However, such dynamic features of *in vivo* development are not replicated in cell production *in vitro*. Cells cannot proliferate or migrate continuously due to the inherent limitations of static culture within the confines of culture vessels. We ask what would happen to cells *in vitro* if cell adhesion were modulated during differentiation.

In this study, we use the cardiac differentiation platform to explore the impact of cell adhesion remodeling during differentiation. We discover that cell dissociation and replating during differentiation significantly alter cell fate and consistently drives mesendodermal progenitors toward cardiomyocytes, bypassing the need for WNT inhibition. The resulting cardiomyocytes are of high purity and mature faster compared to cardiomyocytes differentiated in static culture. This simple manipulation can potentially be applied to the *in vitro* generation of other desired cell types to improve differentiation efficiency and consistency.

## Results

### Cell passaging induces cardiac fate with expanded proliferation potential

**Figure 1:**
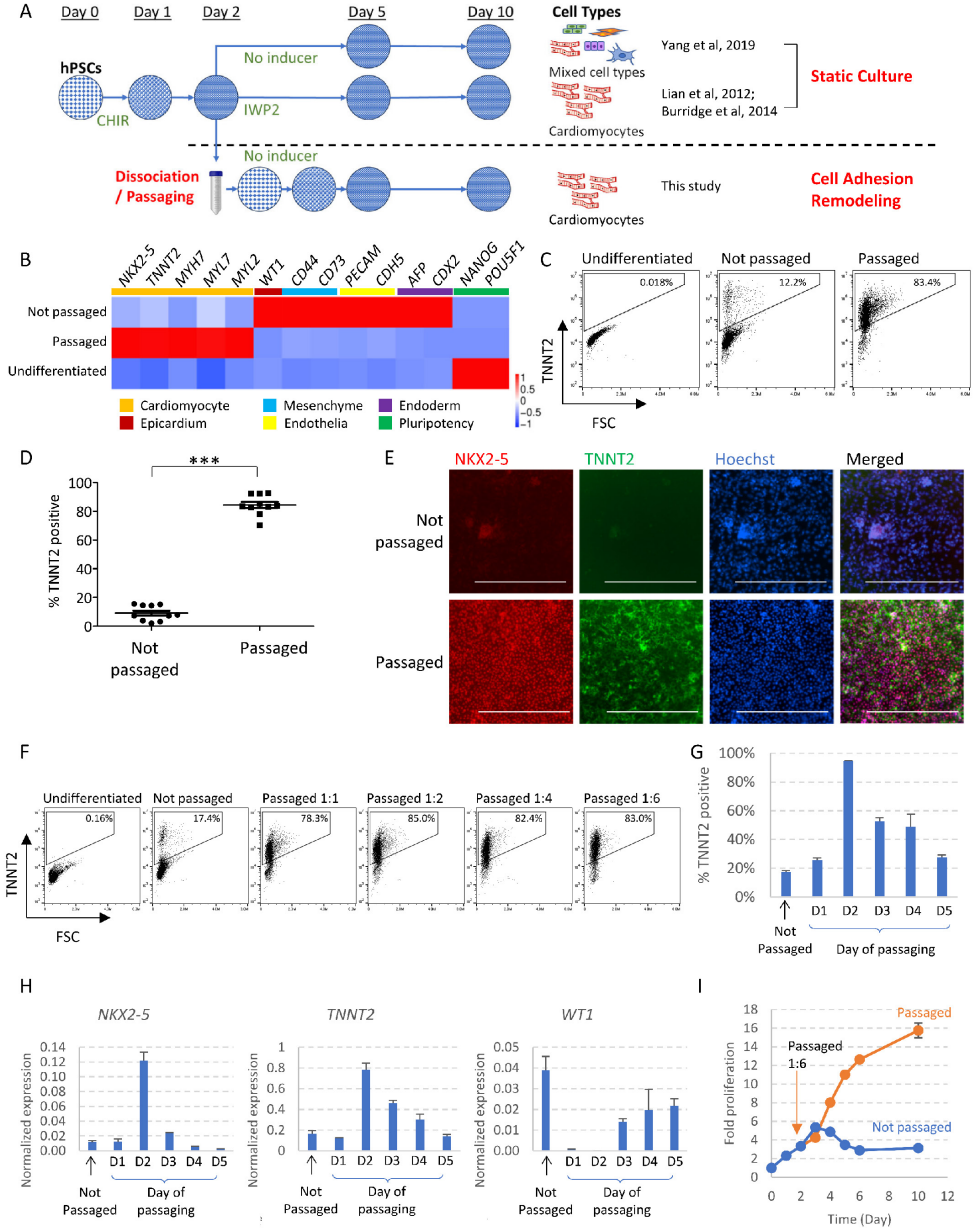
** Cell adhesion remodeling induces cardiac fate with expanded proliferation potential. See also Figure S1 and Supplemental Video 1.** A) Schematic drawing of our differentiation platform in comparison to conventional differentiation in static culture. hPSCs are induced toward mesendoderm progenitors with WNT activator CHIR99021 (5µM in DF medium on Day 0, followed by insulin 1µg/ml in DF medium on Day 1). On conventional differentiation platforms, if no external inducers were applied subsequently, cells kept in static culture will yield a mixture of different cell types (Top); If WNT inhibitor IWP2 was applied, cells will be directed toward cardiomyocyte cell fate (Middle). In this study, we examine the impact of cell adhesion remodeling on cell fate determination (Bottom). Cells are dissociated and passaged onto new culturing surfaces on Day 2 (or other specified time points), and the outcomes of differentiation are examined by Day 10. Cardiomyocytes are induced by passaging without IWP2 or other inducers. B) Heatmap of gene expression levels on Day 10 of differentiation, measured by qPCR (n = 3 biological replicates; Data are representative of three independent experiments). Mesendoderm progenitors derived from H1 cells were allowed to spontaneously differentiate with or without cell dissociation and replating on Day 2 (passaged versus not passaged). Samples were collected on Day 10. Data are normalized to *GAPDH* and heatmap generated using ImageGP (scaled by column) 35. C-D) Percentage of cells expressing TNNT2, measured by FACS analysis on Day 10 of differentiation (Data presented as mean ± SEM, n = 10 independent experiments). ***, p < 0.001 by Student's t-test. E) Immunostaining showing expression of NKX2-5 and TNNT2 on Day 10 of differentiation. Scale bar, 400µm. F) FACS analysis of TNNT2^+^ cells on Day 10. Cells were passaged on Day 2 at different split ratios and compared with static culture. Data are representative of three independent experiments. G) Flow cytometry analysis of TNNT2^+^ cells on Day 10, comparing cells dissociated and replated on different days in the differentiation process. Data presented as mean ± SD of three biological replicates. H) qPCR analysis on Day 10 of differentiation, comparing cells dissociated and replated on different days in the differentiation process. Data were normalized to *GAPDH* and presented as mean ± SD of three biological replicates. I) Total cell count with or without passaging (at a ratio of 1:6) on Day 2 of differentiation, normalized to Day 0 cell count. Data presented as mean ± SD of three biological replicates and are representative of three independent experiments.

Traditionally, differentiation in monolayer format is carried out with cells statically staying in the same wells throughout the differentiation process. For cardiac differentiation, hPSCs were induced to mesendoderm progenitors by WNT activator CHIR99021, and then directed toward cardiomyocytes through WNT inhibition 2, 3. Without specific inducers like WNT inhibitor, mesendoderm progenitors will spontaneously differentiate toward multiple cell types 5 (Figure 1A). In this study, we investigated whether cell fate is affected by passaging cells to new surfaces in the middle of the differentiation process. In contrast to the traditional format, we dissociated and re-plated the differentiating cells on Day 2 of differentiation at a split ratio of 1:4 to 1:6. Following this manipulation, cells re-attached to Matrigel-coated surface, and continued to differentiate (Figure S1A). To our surprise, cell dissociation on Day 2 significantly altered cell fate and directed cells toward cardiomyocytes without the application of any external inducers. Real-time PCR showed greatly enhanced cardiac gene expression and decreased expression of genes in epicardium, mesenchyme, endothelial, liver and intestine cell types following cell passaging (Figure 1B). In comparison, cells that spontaneously differentiated in static culture (not passaged, no pathway modulation) produced a mixture of meso-endodermal cell types (Figure 1B), similar to previous report 5. Cardiomyocyte induction by passaging consistently yielded cardiomyocytes of high purity as shown by flow cytometry (Figure 1C-D) and immunostaining (Figure 1E). In addition to H1 hESCs, passaging also induced cardiomyocytes from H9 hESCs and NL-4 hiPSCs (Figure S1B-C). The impact of passaging on cell fate was replicated at various split ratios, including 1:1 passaging in which the same number of cells were replated into new wells (Figure 1F). These results suggested that cell adhesion remodeling led to cardiac cell fate in the absence of traditional cardiac inducers.

We then examined whether there is a critical time window for cell dissociation to drive cardiac cell fate. When cells were passaged too early on Day 1 or too late on Day 5, there was minimal cardiac promotion effect (Figure 1G-H). Passaging on Day 2, Day 3 or Day 4 promoted cardiac cell fate, suggesting Day 2 to Day 4 is the critical window for cardiac fate commitment. Day 2 was the optimal time point and Day 2-passaging resulted in the highest purity of cardiomyocytes. Cells passaged later (Day 3, 4 or 5) expressed higher levels of *WT1*, suggesting some cells developed into epicardium (Figure 1H). We used Day 2-passaging for the rest of the study unless specified.

We also monitored the impact of passaging on cell number and cell cycle status. In static culture, cell numbers increased slowly in the beginning, and then started to decrease after Day 3, indicating cell death. Cell count stabilized from Day 6 to Day 10, and the total fold increase in cell number is roughly 3-fold compared to Day 0 (Figure 1I). In comparison, following 1:6 passaging, cells continued to proliferate as they differentiated. Starting from Day 4 of differentiation, passaged cells reached significantly higher gross number; By Day 10, the total fold increase reached 16-fold (Figure 1I). Cell cycle profiling by flow cytometry using EdU (5-ethynyl-2´-deoxyuridine) and propidium iodide showed that at the beginning of differentiation, as cell density increased, the proportion of S-phase cells started to decrease significantly by Day 2, and cells became largely quiescent by Day 3. However, with cell passaging on Day 2, the proportion of S-phase cells was significantly elevated on Day 3 (Figure S1D). Taken together, these data suggested that cell adhesion remodeling released the proliferation potential during differentiation while driving cells toward cardiac cell fate.

### Dissociation of cell-matrix adhesion releases cardiac potential during the critical period of cell fate determination

We further analyzed the key factors involved in the passaging process. EDTA was used for cell dissociation in our protocol, so we examined whether it was the EDTA treatment or the cell dissociation itself that led to the enhanced cardiac differentiation. After a brief treatment by EDTA/DPBS, cell-cell contact was disintegrated, but cells still attached to the plate. When EDTA/DPBS was replaced by culture medium without dislocating the cells, cells spread and re-established their contacts within 20 minutes (Figure S1E). Cardiac differentiation was not enhanced in cells that were treated by EDTA but not dislocated from the original adhesion position (Figure S1F-G). In contrast, dislocated cells developed into beating cardiomyocytes even when they were replated at the same density, expressing high levels of cardiac markers *NKX2-5* and *TNNT2*, and lower levels of epicardium marker *WT1* (Figure S1F-G). These results suggested that cell dislocation and rearrangement of cell-ECM contact was the key promoting factor for cardiac cell fate in the absence of cardiac inducers.

### Synergistic signaling modulation maximizes cardiomyocyte production after cell dissociation

**Figure 2:**
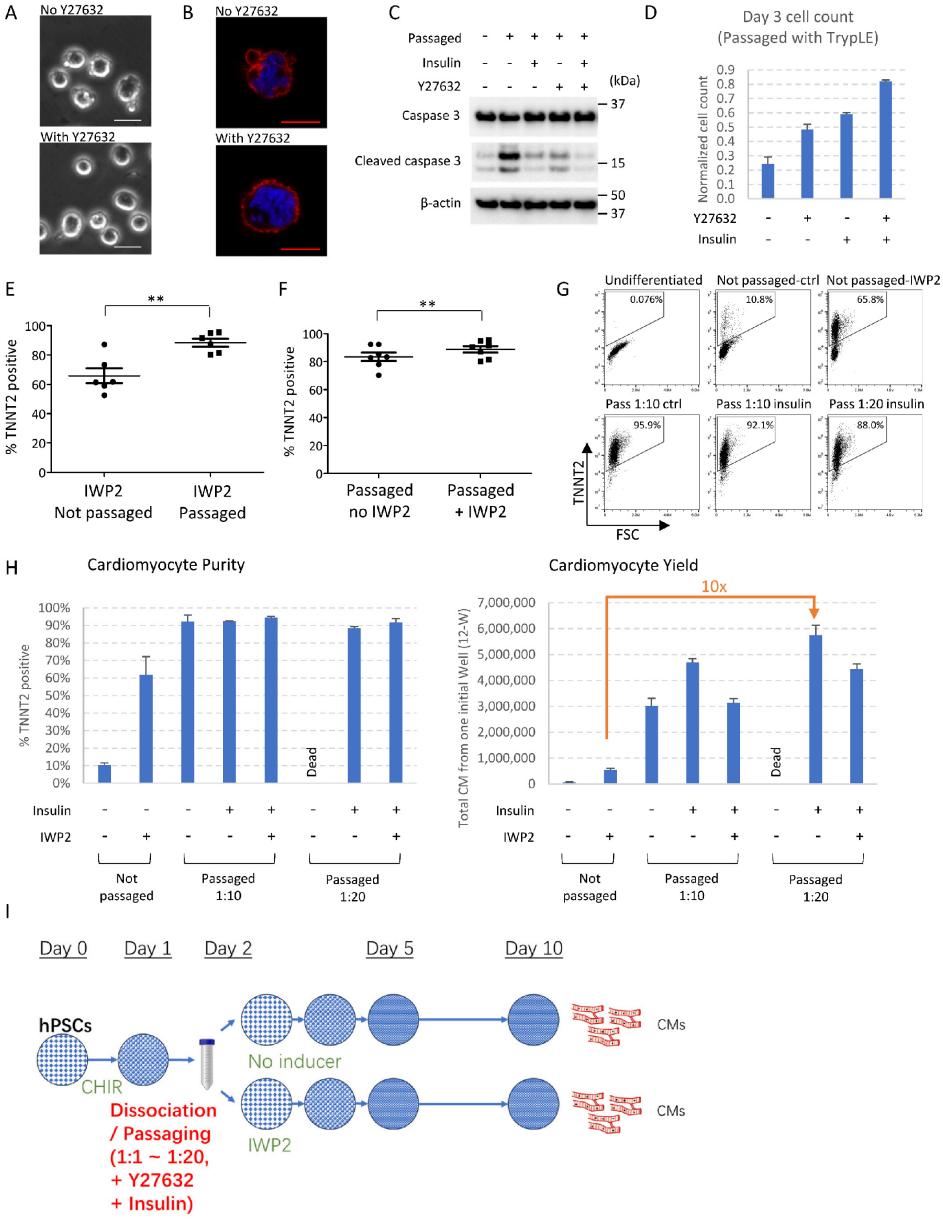
** Synergistic signaling modulation maximizes cardiomyocyte production after cell dissociation. See also Figure S2 and Supplemental Video 2.** A) Phase contrast images of mesendoderm progenitors right after cell dissociation by EDTA on Day 2, in the presence or absence of Y27632. Scale bar, 20µm. B) Phalloidin staining of H1-derived mesendoderm progenitors immediately after cell dissociation with EDTA on Day 2, in the presence or absence of Y27632. Scale bar, 10µm. C) Western blot showing the levels of caspase 3 and cleaved caspase 3 in dissociated mesendoderm progenitors in the presence or absence of insulin and Y27632. Samples were collected 2 hours after cell dissociation by TrypLE Select and replating at 1:6 split ratio. D) Cell count on Day 3, 24 hours after cell dissociation and replating (1:6) on Day 2 using TrypLE Select. Cell numbers were normalized to the number of cells seeded on Day 2. Data presented as mean ± SD of three biological replicates and are representative of three independent experiments. E) FACS analysis of TNNT2^+^ cells on Day 10 of differentiation following IWP2 treatment (applied Day 2-4), with or without passaging (1:6) on Day 2. Data presented as mean ± SEM of 6 independent experiments. **, p < 0.01 by paired t-test. F) FACS analysis of TNNT2^+^ cells on Day 10 of differentiation following passaging (1:6) on Day 2, with or without IWP2 treatment (applied Day 2-4). Data presented as mean ± SEM of 7 independent experiments. **, p < 0.01 by paired t-test. G) FACS analysis of TNNT2^+^ cells on Day 10 of differentiation. Cells were dissociated and replated on Day 2 at split ratios of 1:10 (Pass 1:10) or 1:20 (Pass 1:20) with or without insulin (1µg/ml, applied Day 2~4), and results were compared to static culture. Cells passaged 1:20 without insulin were mostly dead and results were not included. H) Cardiomyocyte purity (% TNNT2^+^) and yield (total cardiomyocytes generated from one initial well on 12-well plate) on Day 10 of differentiation following low-density passaging on Day 2 (split ratios 1:10 or 1:20), with or without insulin (1µg/ml, Day 2-4) or IWP2 treatment (3µM, Day 2-4). Data presented as mean ± SD of three biological replicates and are representative of three independent experiments. I) Schematic drawing of the optimized protocol for cardiomyocyte induction by passaging. CHIR, CHIR99021. The addition of insulin (1µg/ml) is optional at lower split ratios and required at higher split ratios.

ROCK inhibition and insulin are critical for hPSC survival after dissociation 8-10, so we examined the impact of ROCK inhibitor and insulin on mesendoderm progenitors. Without ROCK inhibitor or insulin, some mesendoderm progenitors survived after passaging. However, excessive membrane blebbing was observed in dissociated cells, which was suppressed by ROCK inhibitor Y27632 (Figure 2A-B). Caspase 3 cleavage was elevated in dissociated cells, and was suppressed by insulin and Y27632, suggesting insulin and Y27632 inhibited cell apoptosis during progenitor cell dissociation (Figure 2C). Cell count on Day 3 showed that Y27632 and insulin each suppressed cell death and improved cell survival. These survival-promoting effects were observed both with enzymatic dissociation (using TrypLE Select) (Figure 2D) and with EDTA dissociation (Figure S2A), and were more significant when enzymatic dissociation was used. EDTA dissociation was used for the rest of the study because it had better survival than enzymatic dissociation. We further show that most dissociated cells developed toward cardiac cell fate, regardless of whether Y27632 or insulin was added during passaging (Figure S2B). Cell count and FACS analysis on Day 10 showed that treatment with Y27632 and insulin on the day of dissociation generated higher numbers of cardiomyocytes (Figure S2B-C). These results showed that ROCK inhibitor and insulin can be used to improve cardiomyocyte yield in passaging-induced cardiac differentiation. Based on these findings, Y27632 was added in all subsequent differentiation experiments. Because insulin is known to inhibit cardiac differentiation 5, a low dose (1 µg/ml) was used, and its role under various conditions (different split ratio, with or without WNT inhibitor) was further investigated in subsequent experiments.

As WNT inhibition is widely used for cardiomyocyte induction, we then examined the combined effects of cell dissociation and WNT inhibitor IWP2. Compared to IWP2-induced cardiac differentiation in static culture, cell passaging on Day 2 combined with IWP2 treatment (applied on Day 2-4 of differentiation) produced cardiomyocytes with significantly higher purity and improved consistency (Figure 2E). On the other hand, compared to cell passaging alone, IWP2 treatment after passaging resulted in further improvement of purity (Figure 2F). Day 2 was still the best time point for passaging to drive cardiac induction when IWP2 was applied (Figure S2D). These results indicated that cell dissociation and WNT inhibition synergistically promoted cardiac induction.

We then investigated whether cardiac yield could be further enhanced with a higher split ratio. Our results show that high-purity cardiomyocytes could be induced at a split ratio of 1:10 to 1:20 (Figure 2G). The addition of insulin (1µg/ml) was optional at lower split ratios (1:6 in Figure S2B, 1:10 in Figure 2G-H), but required at higher split ratios (1:20 in Figure 2G-H) to maintain cell survival.

With the optimized protocol, a starting culture of 0.1~0.4 million cells on Day 0 could generate between 5~12 million total cells at the end of a 10-day differentiation after cell passaging on Day 2 (Figure S2E), 4~8 million when combined with IWP2 treatment (Figure S2E). Compared to the traditional method using IWP2 in static culture, passaging-driven differentiation at a split ratio of 1:10 increased cardiomyocyte yield by 8-fold, and 1:20 splitting increased the yield by over 10-fold (Figure 2H). Insulin was required for survival at higher split ratios, and high cardiomyocyte purity could be achieved independent of IWP2 treatment after dissociation (Figure 2H). These results demonstrated the potential of application for passaging-induced cell differentiation.

Given the critical role of cell dissociation and re-adhesion in driving cardiac cell fate, we also examined the effect of different cell culture substrates. Cell dissociation induced cardiac differentiation on multiple kinds of substrates, including Matrigel, E-Cadherin, vitronectin and gelatin (Figure S2F-H). When plated on non-coated surfaces, cell survival was poor, and few cells remained by Day 10 (Figure S2F).

Taken together, high purity cardiomyocytes could be successfully induced by passaging at a wide range of split ratios on various coating materials, which could achieve up to 10-fold increase of cardiomyocyte yield compared to traditional cardiac induction using WNT inhibition in static culture. The optimized induction method is illustrated in Figure 2I.

### Transcriptomic analysis of cardiac induction following cell adhesion remodeling

**Figure 3:**
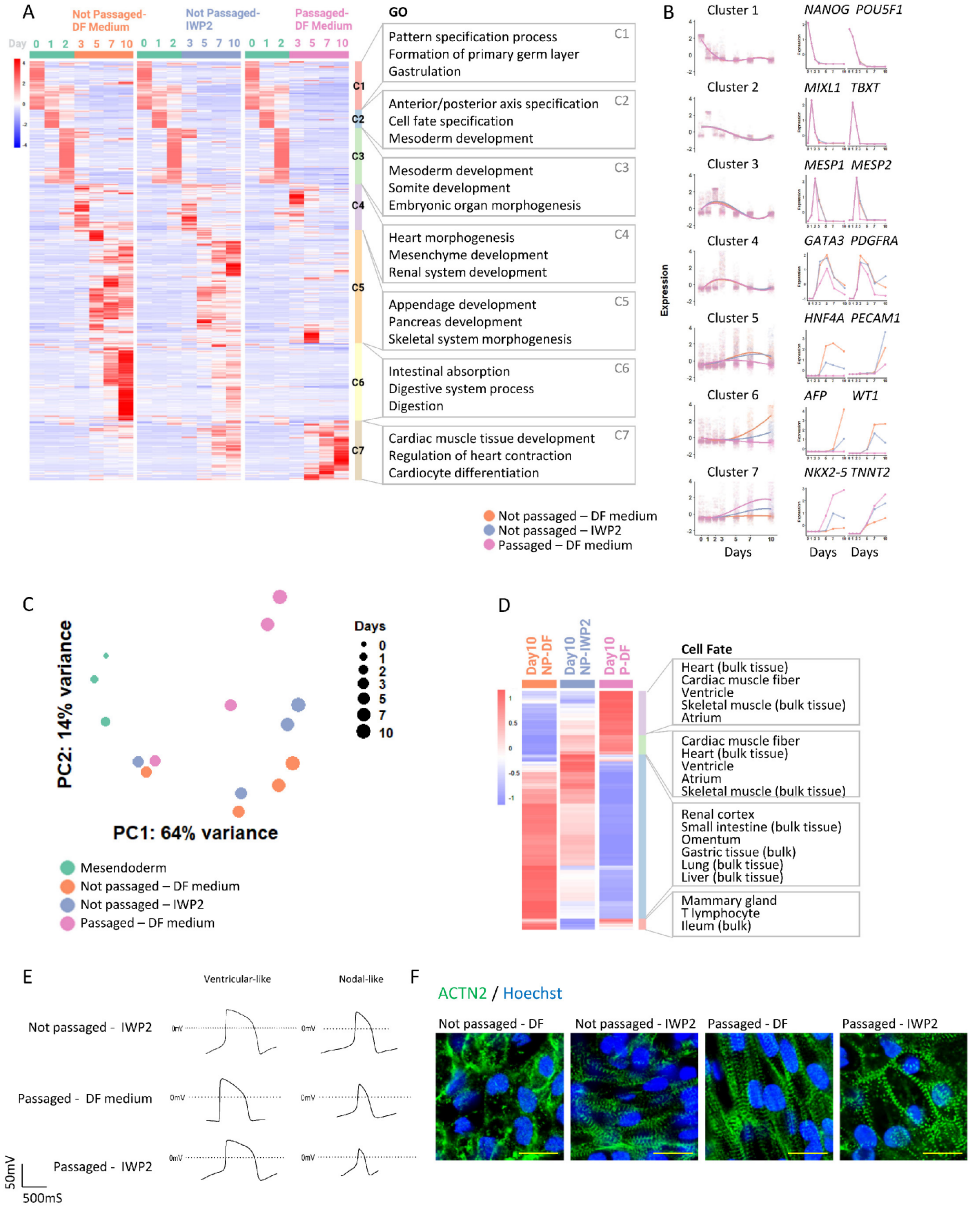
** Transcriptomic analysis of cardiac induction following cell adhesion remodeling. See also Figure S3 and Table S3.** A) Heatmap with hierarchical clustering showing differentially expressed genes (DEGs) at different time points under various treatments (pval < 0.05, logFC > 5). All the groups were subjected to the same treatments from Day 0-2. H1 cells were induced toward mesendoderm progenitors as described in Figure 1A, and then were allowed to spontaneously differentiate without treatment (Not passaged - DF medium), induced by IWP2 treatment (Not passaged - IWP2), or passaged 1:6 on Day 2 and then allowed to differentiate without treatment (Passaged - DF medium). DF medium: DMEM/F12, ascorbic acid, selenite, transferrin, chemically defined lipid, penicillin/streptomycin. Samples were collected for RNA-seq on Day 0, 1, 2, 3, 5, 7 and 10. Gene Ontology (GO) terms associated with each cluster are shown on the right. B) Left: Cubic spline curves for the seven clusters from (A), comparing the three differentiation conditions mentioned above, each point representing an individual gene. Shaded areas represent the 95% confidence interval. Expression levels of all genes were standardized to mean = 0 and SD = 1. Right: Expression of representative genes over time under the three differentiation conditions (computed counts per million, CPM). C) Principal component analysis (PCA) plot illustrating cell differentiation under three conditions. Green dots, differentiation of H1 cells toward mesendoderm progenitors (Day 0-2). Orange, blue and pink dots represent cells under different treatments on Day 3, 5, 7 and 10. Sizes of dots represent days of differentiation. D) Heatmap showing Day 10 DEGs from RNA-seq. Cell type enrichment analysis was performed with Enrichr using ARCHS4_Tissues. E) Representative patch clamp recordings of cardiomyocyte action potentials in cardiomyocytes induced by IWP2, passaging, and combined treatment with both IWP2 and passaging. F) Immunostaining of ACTN2 (green) in Day 20 cells differentiated from H1 under different conditions. Nuclei were stained with Hoechst (blue). Scale bar, 20µm.

To better understand cardiac differentiation following cell adhesion remodeling, we investigated transcriptomic changes through the differentiation process and compared the profile with cardiac differentiation induced by WNT inhibition (Figure 3A). Spontaneous differentiation in static culture (no passaging, no inducer) was included as control. From Day 0 to Day 2, cells were under the same treatment, so the gene expression profiles were shared by the three conditions. Pluripotency markers decreased in the first 2 days (Cluster 1, Figure 3A and 3B); primitive streak and mesoderm progenitor markers emerged on Day 1 and Day 2, and decreased afterwards (Cluster 2). Gene Ontology (GO) enrichment analysis showed that these changes were associated with biological processes of pattern specification, formation of primary germ layer, cell fate specification, and mesoderm development (Figure 3A). We noticed that cardiac progenitor genes such as *MESP1/MESP2* were expressed early, even without cardiac inducers (Cluster 3). Expression of other progenitor markers peaked around Day 3 (Cluster 4).

From Day 3 onward, cell differentiation was directed by WNT inhibitor IWP2 (applied from Day 2 to Day 4) or by passaging (on Day 2). In comparison to the untreated control, cardiac markers, such as *NKX2-5* and *TNNT2* in cluster 7, were elevated by IWP2 from Day 5 to Day 11, and even more so by passaging. Cluster 7 was associated with biological processes of cardiac muscle tissue development and regulation of heart contraction. The expression of other lineage markers rose in control cells around the same time period; their expression levels were lower in IWP2-treated cells, and much lower in cells that were passaged (Cluster 5 and 6). These data suggested that intermediate passaging promoted cardiac cell fate and suppressed other meso-endoderm fates which would have emerged spontaneously without treatment.

We then compared the gene expression profiles of cells induced by IWP2 versus those induced by passaging. Principal component analysis (PCA) showed different trajectories of cell differentiation under IWP2-treated, passaged and untreated conditions (Figure 3C). Cell fate analysis of differentially expressed genes (DEGs) on Day 10 showed enrichment of cardiac cell fates in the passaged cell population (Figure 3D). These data confirmed that intermediate passaging drove cell fate toward cardiomyocytes without external inducers.

We then analyzed the functional characteristics of the cardiomyocytes induced by passaging. Cardiomyocytes induced by IWP2, passaging, and IWP2 plus passaging all demonstrated typical cardiomyocyte electrophysiology profiles by patch clamp measurement (Figure 3E) and by field potential measurement on Maestro microelectrode array (MEA) system (Figure S3A). Immunostaining of α-actinin in Day-20 cardiomyocytes showed well-organized myofibril structures in all three types of cardiomyocytes, indicating proper sarcomeric organization (Figure 3F). These data showed that cardiomyocytes generated by cell adhesion remodeling displayed typical cardiomyocyte characteristics.

### Cell adhesion remodeling regulates AMPK and AKT signaling through the integrin pathway

**Figure 4:**
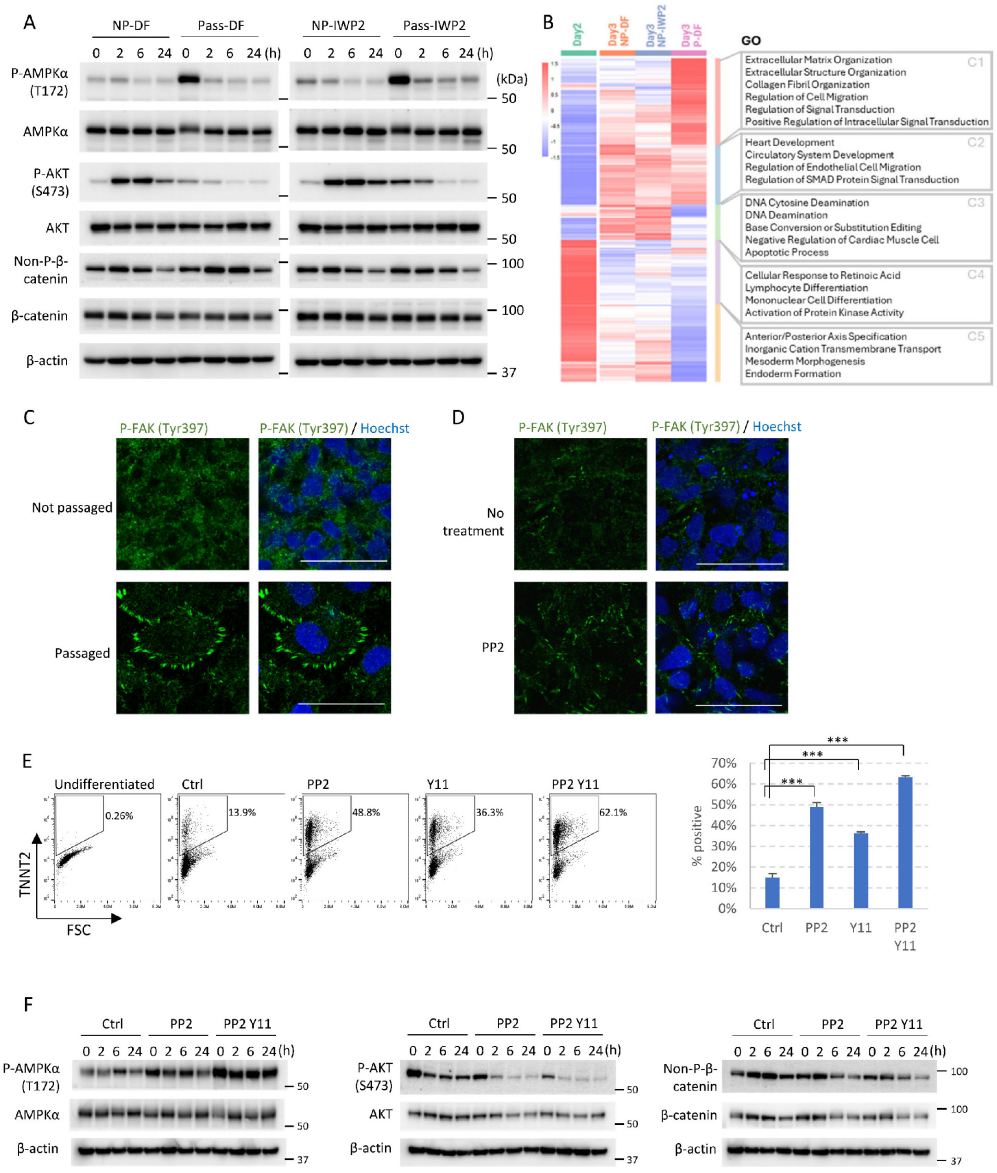
** Cell adhesion remodeling regulates AMPK and AKT signaling through the integrin pathway. See also Figure S4.** A) Western blot showing levels of phosphorylated AMPKα (T172), total AMPKα, phosphorylated AKT (S473), total AKT, non-phosphorylated (active) β-catenin and total β-catenin. Mesendoderm progenitors (derived from H1 cells as shown in Figure 1A) on Day 2 of differentiation were either passaged using EDTA or not passaged (left in the same well and changed to fresh DF medium), with or without IWP2 treatment. Samples were collected right after manipulation (0 hr) and after 2, 6, 24 hours. NP-DF, not passaged and no treatment. Pass-DF, passaged and no treatment. NP-IWP2, not passaged, treated with IWP2 (3µM). Pass-IWP2, passaged on Day 2 and treated with IWP2 (Day 2-4, 3µM). β-actin was used as loading control. B) Heatmap showing Day 2-3 DEGs from RNA-seq. GO terms associated with each cluster are shown on the right. C) Immunostaining of phosphorylated FAK (Tyr397, green) in passaged versus non-passaged cells. H1 cells were induced to mesendoderm progenitors as described in Figure 1A. Cells were fixed for immunostaining two hours after medium change (non-passaged well, DF medium) or passaging (passaged well, DF medium) on Day 2. Nuclei were stained with Hoechst (blue). Scale bar, 50µm. D) Immunostaining of phosphorylated FAK (Tyr397, green) after SRC inhibitor treatment. H1 cells were induced to mesendoderm progenitors. Cells were treated with or without PP2 (5µM) on Day 2 for 24 hours in DF medium, cultured in DF medium for another day, and fixed for immunostaining. Nuclei were stained with Hoechst (blue). Scale bar, 50µm. E) Flow cytometry analysis of TNNT2^+^ cells on Day 10 following treatment with PP2, Y11 or both on Day 2. Data presented as mean ± SD of three biological replicates. ***, p < 0.001 compared to control (One-Way ANOVA with Dunnett's Multiple Comparison Test). F) Western blot showing levels of phosphorylated AMPKα (T172), total AMPKα, phosphorylated AKT (S473), total AKT, non-phosphorylated (active) β-catenin and total β-catenin. Mesendoderm progenitors (derived from H1 cells as shown in Figure 1A) on Day 2 of differentiation were treated with PP2 (5µM) or PP2 combined with Y11 (10µM) and compared to control (Ctrl, fresh DF medium only). Samples were collected right after manipulation (0 hr) and after 2, 6, 24 hours. β-actin was used as loading control.

To investigate the mechanisms of passaging-driven cardiac differentiation, we analyzed the major signaling pathways that are known to be important for cardiac cell fate determination, including the AMPK pathway 11, PI3K/AKT pathway 5, and canonical WNT signaling 4. Western blot analysis following cell dissociation and replating on Day 2 showed that compared to non-passaged control, cell passaging significantly up-regulated phosphorylation of AMPK (T172) and suppressed AKT phosphorylation (S473) during the 24-hour period following passaging (Figure 4A). Immunostaining results are consistent with these observations (Figure S4A-B). Activation of AMPK pathway and inhibition of AKT signaling are both well known to drive cardiac cell fate 5, 11. In comparison, the level of non-phosphorylated (active) β-catenin was not significantly changed (Figure 4A), but the subcellular localization shifted toward the membrane on immunostaining (Figure S4C). When CHIR99021 was applied after cell passaging (Day 2-4), cardiac differentiation was suppressed in a dose-dependent manner, further confirmed that cell passaging induces cardiomyocytes partially through the WNT pathway (Figure S4D). Taken together, these data suggested that cell adhesion remodeling altered the activities of key signaling pathways in cells, which in turn affected cell fate decisions and allowed cardiac differentiation independent of exogenous WNT inhibition.

How does cell adhesion remodeling lead to these signaling changes? To answer this question, we analyzed the transcriptomic changes on Day 3 in passaged versus non-passaged cells by RNA sequencing (Figure 4B). Compared to static culture, passaging on Day 2 significantly elevated gene expression in extracellular matrix organization, collagen fibril organization, and regulation of signal transduction (Figure 4B, Cluster 1). Genes down-regulated by passaging included those involved in the apoptotic process, activation of protein kinase activity, and endoderm formation (Figure 4B, Cluster 3-5), suggesting cell dissociation rearranged cell-matrix interactions and altered signal transduction. As the integrin pathway is crucial for cell-ECM interaction, we hypothesized that the integrin pathway may be involved in dissociation-driven cell fate changes.

To test the above hypothesis, we examined the focal adhesions in passaged versus non-passaged cells, because focal adhesions connect integrins to the actin cytoskeleton and serve as hubs for signal transduction. Immunostaining of phosphorylated FAK (Y397) showed that cell passaging on Day 2 significantly elevated the number of focal adhesions on the cell periphery (Figure 4C). This phenotype can be mimicked in non-passaged cells by treatment with PP2, an inhibitor of Src family kinases (Figure 4D). We then examined whether modulation of integrin signaling can induce cardiac cell fate in non-passaged cells. Indeed, treatment with PP2 on Day 2 can mimic the effect of cell passaging and promote differentiation toward cardiac cell fate, and the effect can be further improved when PP2 is combined with FAK1 inhibitor Y11 (Figure 4E and S4E). We further analyzed the downstream signaling pathways, and observed similar patterns of signaling changes. PP2 treatment elevated AMPK phosphorylation, and suppressed AKT phosphorylation. The effects are further enhanced when PP2 is combined with Y11 (Figure 4F). Taken together, these results suggest that the integrin pathway and its downstream signal transduction events are critically involved in cell fate determination.

### Cell adhesion remodeling accelerates cardiac fate determination and maturation

**Figure 5:**
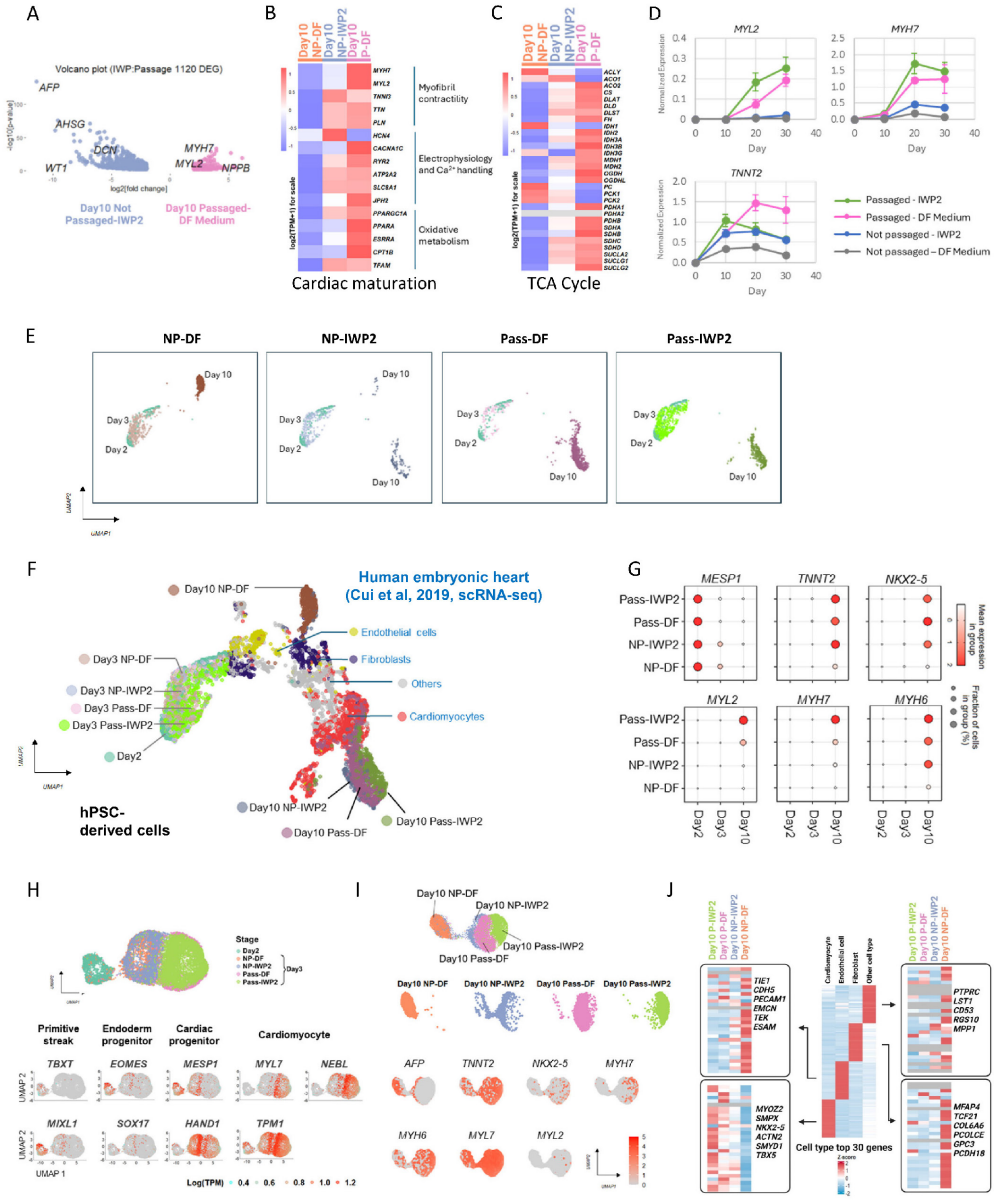
** Cell adhesion remodeling accelerates cardiac fate determination and maturation. See also Figure S5.** A) Volcano plot showing DEGs between Day 10 cardiomyocytes induced by passaging and by IWP2 treatment. The x-axis corresponds to fold changes on a log2 scale. The y-axis corresponds to the P-value measured on a log10 scale. B) Heatmap showing the expression of cardiac maturation marker genes on Day 10 in cells differentiated under no treatment (NP-DF), treated with IWP2 (NP-IWP2), or passaged on Day 2 (P-DF). C) Heatmap showing the expression of TCA cycle genes on Day 10. D) Expression of *MYL2*, *MYH7* and *TNNT2* in H1-derived cardiomyocytes induced by IWP2 (Not passaged-IWP2), passaging (Passaged-DF Medium), or both (Passaged-IWP2), measured by qPCR on Day 10, 20 and 30 of differentiation. Control wells (Not passaged-DF medium) were kept in DF medium from Day 2 without passaging or IWP2. IWP2 treatment was applied from Day 2 to Day 4. Passaging was performed on Day 2. Cells were maintained in DF medium with daily medium change. Data presented as mean ± SD of three biological replicates and are representative of three independent experiments. E) Uniform Manifold Approximation and Projection (UMAP) plots generated with top 2000 variable features from single cell RNA sequencing of H1-derived cells on Day 2, 3, 10 of differentiation. H1 cells were induced to mesendoderm progenitors as described in Figure 1A, then cultured under four conditions: DF medium only with daily medium change (NP-DF), IWP2 treatment (NP-IWP2, 3µM applied Day 2-4), passaged on Day 2, then cultured in DF medium (Pass-DF), passaged on Day 2, and treated with IWP2 (Pass-IWP2, 3µM applied Day 2-4). F) Overlaid UMAP plot of two scRNA-Seq datasets, human embryonic heart (GSE106118) 14 and H1-derived cells (on Day 2, 3, 10 of differentiation). Colors represent different treatments / time points in our data, and different cell types in human embryonic heart data. G) Dot plot of scRNA-seq data showing expression patterns of representative genes in cells differentiated under different treatments. Dot sizes represent the fraction of cells within each group. The color shades of the dots represent the mean expression level of genes. H) Analysis of Day 2-3 scRNA-seq data. Top: UMAP plot, Colors indicate different treatments. Bottom: Feature plots illustrating the expression of marker genes for primitive streak, endoderm progenitor, cardiac progenitor and cardiomyocytes on Day 2-3. Color scales represent log2(TPM+1). I) Analysis of Day 10 scRNA-seq data. Top: UMAP plot, Colors indicate different treatments. Middle: Faceted view of the distribution of cells from different treatments in UMAP. Bottom: Feature plots illustrating the expression of marker genes on Day 10. Color scales represent log2(TPM+1). J) Middle: Heatmap displaying the Z-score-scaled average expression levels of the top 30 DEGs in human embryonic heart cell types. Left and right: Heatmaps showing the expression of top 30 DEGs from human embryonic heart cells in hPSC-derived cells generated under different treatments, based on Day 10 scRNA-seq results. Gray indicates expression values of 0.

We then compared the cardiomyocytes generated through cell adhesion remodeling with those generated using WNT inhibition, which is the mainstream method for cardiac induction. During the differentiation process, passaging on Day 2 led to a more rapid decrease of cardiac progenitor *MESP1* and *MESP2* gene expression, and cardiac markers emerged early when cells were passaged (Figure 3A-B). RNA-seq analysis of DEGs between IWP2- and dissociation-induced cardiomyocytes on Day 10 showed up-regulation of multiple cardiac maturation markers by passaging, such as *MYH7*, *MYL2* and *NPPB* (Figure 5A). We then compared the expression of reported cardiac maturation markers 12, 13 between the two cell types, and observed an overall up-regulation of maturation markers and TCA cycle components in passaging-induced cardiomyocytes compared to static culture on Day 10 (Figure 5B-C).

To confirm the impact of passaging on maturation, we performed additional RNA-seq analysis on cells induced by IWP2, passaging or both on Day 12 and Day 16 of differentiation. Compared to static culture, cardiac maturation markers and TCA cycle genes were up-regulated by passaging with or without IWP2 treatment (Figure S5A-B). The elevation of *MYL2* and *MYH7* were confirmed by real-time PCR in Day 10, 20 and 30 samples (Figure 5D). Compared to IWP2-induced cardiomyocytes in static culture, by Day 30 of differentiation, *MYL2* levels were elevated over 9-fold by passaging, and 12-fold by passaging combined with IWP2 treatment. *MYH7* levels were elevated 3-fold by passaging and 4-fold by passaging plus IWP2. These results indicate that cardiac induction by passaging accelerates the differentiation process and promotes cardiomyocyte maturation compared to differentiation in static culture.

To further characterize passaging-induced cardiomyocytes, we then conducted single cell RNA sequencing (scRNA-Seq) to map the transcriptional landscapes of passaged versus non-passaged cells on Day 2, 3 and 10 of differentiation, in the presence or absence of IWP2 (Figure 5E). After quality control filtering, 24,000 cells were retained for transcriptome analysis. For cell type identification, cells in each sample were projected into an adjusted 2D space using Uniform Manifold Approximation and Projection (UMAP) and overlaid with published data set of human embryonic heart cells 14 for comparison (Figure 5F). Four major cell types were annotated in the published *in vivo* data, including cardiomyocytes, fibroblast-like cells, endothelial cells, and other cell types. In our data, hPSC-derived cells clustered according to the differentiation stage, and grouped into two distinct populations on Day 10 (Figure 5E-F). Cells that differentiated in static culture without treatment (NP-DF) were located next to fibroblasts on the UMAP plot. Cells induced by IWP2 (NP-IWP2) or passaging (Pass-DF, Pass-IWP2) distributed to the cardiomyocyte region, and passaging-induced cells located more closely to *in vivo* cardiomyocyte clusters compared to IWP2-induced cells from static culture (Figure 5F). Through the differentiation process, early cardiac progenitor *MESP1* was highly expressed on Day 2, then the expression level declined in all conditions, and faster decline was seen in passaged cells (Figure 5G). Cardiac marker genes, including *TNNT2*, *NKX2-5*, *MYL2, MYH7*, and *MYH6*, were induced by either IWP2 treatment or cell dissociation, or both, and the combination of IWP2 treatment and passaging generated cells with highest maturation marker gene expression (Figure 5G). These observations are consistent with bulk RNA sequencing results. Based on the developmental stages of human embryonic heart data, passaged cells on Day 10 of differentiation distributed closely to embryonic cardiomyocytes between week 5-9 (Figure S5C). For better visualization of the differentiation process, we then used Monocle 3 to perform second-level dimensional reduction of Day 2-3 data (Figure 5H). Primitive streak markers *TBXT* and *MIXL1* were expressed on Day 2. On Day 3, passaged and non-passaged cells showed distinct profiles. Passaged cells expressed lower levels of endoderm progenitor markers, including *EOMES* and *SOX17*. Expression level of procardiac gene *MESP1* was high in Day 2 cells and lower in passaged cells by Day 3, suggesting faster progression to the next stage. *HAND1* was expressed in both passaged and non-passaged cells on Day 3. Cardiomyocyte markers *MYL7*, *TPM1* and *NEBL* emerged early and were expressed at higher levels in passaged cells compared to non-passaged ones on Day 3 (Figure 5H).

Analysis of gene expression on Day 10 showed that hepatocyte marker *AFP* was expressed in non-treated static cells and some IWP2-induced cells (Figure 5I). IWP2- or passaging-induced cells expressed cardiomyocyte markers, including *TNNT2, NKX2-5, MYH7*, *MYH6, MYL7* and *MYL2*, and the overall levels are higher in passaged cells (Figure 5I). Expression levels of additional marker genes from the four cell types in human embryonic heart 14 were analyzed, and the levels of top 30 genes are shown in Figure 5J. Cardiomyocyte markers were enriched in passaging-induced cells, and even more so when passaging was combined with IWP2 treatment. Markers of endothelial cells, fibroblasts and other cell types were enriched in spontaneously differentiated cells in static culture (Figure 5I).

For comparison, the same set of marker genes from human embryonic heart were also used to analyze bulk RNA-seq data, and similar trends were observed (Figure S5D).

Taken together, these data demonstrated that intermediate passaging induced cardiomyocytes in 10 days and suppressed differentiation toward non-cardiac cell types; passaging-induced cells showed gene expression profiles that better matched human embryonic cardiomyocytes, and expressed cardiac maturation markers at higher levels.

In summary, we report here a useful method to redirect cell fate through cell adhesion remodeling. This simple protocol generates cardiomyocytes independent of WNT inhibitor, improves cardiomyocyte yield by up to 10-fold and accelerates cardiomyocyte maturation. Similar manipulations could be applied to other differentiation platforms for optimized production of various cell types, providing a valuable tool for research and clinical applications.

## Discussion

Production of high-purity cardiomyocytes in large quantities is the key for clinical applications. In this study, we discovered that remodeling of cell adhesion during differentiation promotes cardiomyocyte induction from mesendoderm progenitors with significantly enhanced yield. This finding not only offers a low cost and highly efficient method for cardiomyocyte production, but also highlights an interesting phenomenon in hPSC differentiation that cell dissociation at critical points of differentiation redirects cell fate while maintaining the proliferation potential.

The extracellular matrix (ECM) is an essential component of stem cell niche and plays important roles in cell fate determination. In monkey ESCs, highly adhesive ECM substrates like collagen were shown to suppress differentiation, while the less adhesive Matrigel directed cells toward endoderm cell fate, and cells cultured on non-adhesive substrate (agarose) or in suspension displayed elevated cardiac gene expression 15. Matrices of different stiffness were reported to direct mesenchymal stem cells toward different cell fates 16. In static culture, most cells cannot migrate due to high confluence during differentiation, and various cell types emerge from mesendoderm progenitor cells 5. In this study, we show that cell dissociation at critical time points allows cells to re-arrange cell adhesion and migrate, and significantly promotes the generation of cardiomyocytes with high purity. This phenomenon suggests that cell adhesion could have profound influence on cell fate determination.

The crosstalk between cell adhesion and intracellular signaling is well reported. Mechanical strain was reported to inhibit hESC differentiation through the TGFβ/Nodal/Activin signaling pathway 17. ECM protein fibronectin plays an essential role in the development of the asymmetric body plan in mice through activation of Nodal pathway and phosphorylation of Smad2/3 18. Degradation of hyaluronan, a key ECM component, leads to mitochondrial stress through TGFβ pathway 19. To realize this kind of crosstalk between extracellular stimuli and cellular responses, the integrin family of transmembrane receptors play a key role. When activated by extracellular cues, integrins recruit intracellular proteins and regulate multiple intracellular processes, including control of cell migration and survival through FAK/SRC complex, and promotion of cell proliferation through MAPK/ERK and PI3K/AKT 20. Engagement of integrin receptors with the ECM can directly activate Erk, Jnk and p38 MAP kinases, several Rho-family GTPases, and promote the efficient activation of receptor tyrosine kinases 21. On our differentiation platform, we observed a significant decrease in AKT phosphorylation, along with elevated AMPK phosphorylation following the passaging of mesendoderm progenitors, likely mediated by changes in integrin signaling. The impact of cell passaging on cell fate and on key signaling pathways can be mimicked by the inhibition of Src-family tyrosine kinase or focal adhesion kinase, implying how integrin signaling is involved in the cell fate determination process.

During embryogenesis, cells undergo constant movements and rearrangements, and have access to adequate space and nutrients for proliferation. Upregulation of gene expression related to cell migration and ECM organization is a key feature in early human embryonic heart development 14. In contrast, in commonly used *in vitro* differentiation platforms, cells are confined in the same well for prolonged periods of time, and medium is changed on a fixed schedule. Such conditions lead to depletion of resources and contact inhibition due to limited space. By dissociating cells and re-plating them during the critical period of cell fate commitment, our protocol introduces free space, ample nutrients, and reshuffling of cell-cell / cell-ECM contact. This method elevates the expression of functional cardiac genes such as MYH7 and MYL2. More study is necessary to explore how these genes are regulated through cell adhesion during cell fate specification. It is plausible that applying cell adhesion remodeling to other differentiation platforms may also provide a new dimension and help generate previously hard-to-obtain cell types.

Insulin is typically avoided in early cardiac differentiation 3 due to its inhibitory effect on cardiac cell fate. It is often used after Day 7, when cells are already committed to cardiomyocytes, and the commonly used working concentration is 10µg/ml. In our previous study, we found that insulin is important for survival of hESCs after dissociation 9. In this study, we examined its impact on differentiating cells, and also observed enhanced cell survival with insulin treatment. To take advantage of its survival-promoting effect while maintaining cardiac differentiation, we used a low concentration of insulin (1µg/ml). Our results show that insulin significantly enhanced cell survival after passaging (Figure 2D and S2A). When cells are passaged at lower split ratios such as 1:6, addition of insulin led to a slight decrease in purity (Figure S2B) along with an increase in cardiac yield (Figure S2C). In comparison, at higher split ratio such as 1:20, insulin is required for cell survival after passaging, and most cells will die after passaging without insulin (Figure 2H). Overall, successful cardiac differentiation with > 80% TNNT2^+^ cells can be achieved with the use of low-concentration insulin.

Our differentiation platform has several unique features. Cell passaging allows significant expansion of cell number (Figure 1I) and increases the yield of cardiomyocyte production by up to 10-fold compared to IWP2-driven differentiation in static culture (Figure 2H). Compared to cardiomyocyte production in bioreactors 22-25, the cell yield on Day 10 is usually less than 2-fold of the cell input on Day 0 in bioreactors, indicating very limited proliferation. On our platform, cells proliferate more than 10-fold from Day 0 to Day 10, and cell fate determination takes place during continued cell proliferation (Figure 1I). Our RNA-seq data show that passaging-induced cardiomyocytes express higher levels of maturation markers, and the gene expression profile more closely resembles that of embryonic cardiomyocytes compared to the conventional IWP2 method. Cardiomyocyte generation through WNT inhibition is significantly affected by experimental variables like cell plating density and growth rate, leading to batch-to-batch variations. The passaging method, in comparison, consistently produces high-purity cardiomyocytes at a wide range of passaging densities (Figure 1F). It is plausible that a basal level of endogenous signaling normally guides cells to a diverse cell fate in static culture, and this level fluctuates with culture conditions. With the remodeling of cell adhesion and the modulation of key signaling pathways by integrin and its associated proteins, the endogenous signaling is interrupted, and the combined effects of AMPK activation and AKT inhibition leads to generation of cardiomyocytes.

Taken together, we report a method for redirecting cell fate through cell adhesion remodeling. This offers a simple and cost-effective protocol for cardiomyocyte production, and a new angle of cell fate manipulation for clinical and research applications.

## Methods

### Key resources table

### Availability of data and material

RNA-seq data have been deposited in GEO and are publicly available as of the date of publication. The accession numbers are listed in the key resources table.

Requests for resources, reagents and further information should be directed to and will be fulfilled by the lead contact, Guokai Chen (GuokaiChen@um.edu.mo).

### Maintenance of hPSCs

Human embryonic stem cells (hESCs) H1 and H9 and induced pluripotent stem cells (iPSCs) NL-4 were cultured on Matrigel-coated plates in home-made E8 medium 26. Cells were passaged every three days using EDTA method at splitting ratios between 1:6 ~ 1:12 in the presence of ROCK inhibitor Y27632 (5 µM). E8 medium was changed daily. Cells were used for experiments between passage number 30-50 and were routinely tested for mycoplasma, sterility and pluripotency. The use of hPSCs for research was approved by the Institutional Review Board at the University of Macau. All experiments were conducted using H1 cells, unless otherwise specified.

### Differentiation

For differentiation toward mesendoderm progenitors, 70% confluent hPSCs were passaged 1:6 into 12-well plates two days before the start of differentiation as described above and maintained in E8 medium. On Day 0 of differentiation, cells were switched to DF medium (DMEM/F12, ascorbic acid, selenite, transferrin, chemically defined lipid, penicillin/streptomycin) and treated with CHIR-99021 (5 µM), followed by DF medium and insulin (1 µg/ml) on Day 1.

For non-passaged protocol, mesendoderm progenitors were allowed to differentiate in DF medium either spontaneously (without other supplements) or in the presence of IWP2 (3 µM, applied Day 2-4 in DF medium). From Day 5 and onward, cells were cultured in DF medium without additional supplements until sample collection between Day 9-12. Medium was changed daily.

For passaging-driven differentiation, mesendoderm progenitors were dissociated and replated on Day 2 of differentiation (unless otherwise specified). Briefly, cells in 12-well plates were rinsed with DPBS/EDTA (0.5 mM) and incubated in DPBS/EDTA for 5 minutes. After removal of DPBS/EDTA solution, cells were resuspended in DF medium (with or without Y27632 or insulin as described in the manuscript) and plated into new matrigel-coated wells at specified splitting ratios (ratios between 1:1 ~ 1:20 were tested). Cells were then cultured in DF medium until sample collection between Day 9-12. Medium was changed daily. Other treatments were specified if applied.

### Flow cytometry

For FACS analysis, cells were treated with TrypLE Select for 10 minutes and collected as single cells. 5% bovine serum albumin (BSA) was added to neutralize the enzyme. Cells were fixed in 1% paraformaldehyde in 1xPBS for 10 minutes at 37°C, washed, and permeabilized in 90% methanol, 1xPBS for 30 minutes on ice. After blocking with FACS buffer (1% BSA, 0.05% triton X-100, 1xPBS), cells were incubated with primary antibodies (2 hours at room temperature or 4 °C overnight), washed, incubated with fluorescent secondary antibodies, washed again, and resuspended in 1xPBS for analysis by Cytoflex S flow cytometer (Beckman Coulter).

### Cell cycle analysis

Cell cycle status was analyzed using Click-iT® Plus EdU Flow Cytometry Assay Kit following manufacturer's instructions. Briefly, cells at different time points of differentiation were incubated with 10 µM Click-iT® EdU for 30 minutes, harvested using TrypLE Select, then fixed and permeabilized following protocol from the kit. After the Click-iT® reaction, cells were washed, stained with propidium iodide (PI), and then analyzed on flow cytometer.

### Immunostaining

Cells were fixed using 4% paraformaldehyde, 1xPBS, washed, and permeabilized in 0.5% Triton X-100, 1xPBS. After blocking with 1% BSA, 1xPBS, cells were incubated with primary antibodies at 4 °C overnight, washed, and incubated with fluorescent secondary antibodies for one hour in the dark. Hoechst was used for nuclear staining.

### Microscopy

Phase contrast images of cells were taken on on EVOS® FL Auto Imaging System (Thermo Fisher). Fluorescent images were taken on EVOS® FL Auto Imaging System (Thermo Fisher) and Nikon A1R confocal microscope.

### Western blot

For western blotting, cells were lysed in-well using 2xLaemmli Buffer supplemented with protease inhibitor cocktail and phosphatase inhibitors. Protein concentrations of the lysates were measured using BCA assay, and 30µg total proteins were loaded for SDS-PAGE gel. After the gel run, proteins were transferred to PVDF membrane. The blot was blocked in 5% milk, 1xTBST, followed by incubation with primary and secondary (HRP-conjugated) antibodies diluted in 1% BSA, 1xTBST. Chemiluminescence was generated using SuperSignal™ West Pico PLUS Chemiluminescent Substrate or SuperSignal™ West Dura Extended Duration substrate and detected using ChemiDoc MP Imaging System (Bio-Rad). β-actin was used as loading control.

### Quantitative PCR

Total RNA was extracted using RNAiso Plus reagent (Takara) following kit instructions. cDNA was generated from 1µg total RNA using High-Capacity cDNA Reverse Transcription Kit (Applied Biosystems). Quantitative PCR was carried out using TB Green Premix Ex Taq (Takara) on Applied Biosystems ViiA™ 7 Real-Time PCR System. Expression levels were normalized to *GAPDH*.

### Bulk RNA-seq

For bulk RNA sequencing, RNA was harvested using RNAiso Plus (Takara). 1 μg total RNA was used for library preparation. cDNA synthesis was carried out using NEBNext® Single Cell/Low Input cDNA Synthesis & Amplification Module. Libraries were prepared using SMRTbell® prep kit 3.0 following standard protocol.

Sequence reads were trimmed to remove adaptor sequence/low-quality sequence using Cutadapt, and clean data were aligned to reference genome by Hisat2 (v2.0.1). Read count extraction and normalization were performed by HTSeq (v0.6.1). Differential expression analysis was implemented using the DESeq2 Bioconductor package with adjusted p-val < 0.05 as cutoff. Variance-stabilizing transformation (VST) was used to process the global read counts. Hierarchical clustering was performed by Hclust. Gene Ontology (GO) enrichment analysis was carried out by clusterProfiler 27, 28. Cell type enrichment analysis was performed using Enrichr 29-31. For data visualization, we utilized the ggplot2 and pheatmap packages.

### scRNA-seq

For single-cell RNA sequencing, samples were collected on Day 2 (prior to passaging / treatment), Day 3 and Day 10 of differentiation. Cells were harvested and encapsulated using the Nadia system (Dolomite Bio). After reverse transcription and PCR amplification, 800 STAMPs were purified and used for library preparation. Tagmentation of cDNA was carried out with Nextera XT Kit.

After sequencing on Illumina Hiseq PE150 platform, the raw paired-end reads underwent trimming and filtering using the Drop-seq_tools-2.5.3. Subsequently, alignment to the human genome GRCh38 (hg38) was carried out by STAR 32. The default threshold of 200 genes per cell was used as the minimum requirement. Further filtration steps were applied to generate the cell expression matrix. Gene expression levels were quantified as transcripts per million (TPM) across all single-cell datasets. To enhance comparability, TPM values were converted to log2(TPM+1). These transformed values were utilized as input for downstream analyses using *Seurat* 33 and *Monocle 3* 34. The IntegrateData function from Seurat was used to integrate published human embryonic heart cell dataset 14 and our scRNA-seq data, with the cell counts in our data downsized to 3000 to match the *in vivo* dataset. The function FindAllMarkers was used to identify the DEGs in each cell-type (logFC = 0.25, min.pct = 0.01). The specific expression patterns in dot plots, heatmaps and feature plots were generated using the scRNAtoolVis package (version 0.0.5).

### Electrophysiology

Whole-cell patch clamping was carried out in the current clamp mode using Axon Axopatch 200B Microelectrode Amplifier following standard procedure. Field potentials of cardiomyocytes were measured in 48-well multi-electrode array (MEA) on Maestro 768D MEA system (Axion BioSystems), and data were analyzed in AXIS software using Statistics Compiler.

### Statistical analysis

Data are presented as mean±SD of three independent experiments unless otherwise specified. Statistical significance was determined using Student's t-test or One-Way ANOVA with Dunnett's Multiple Comparison Test.

## Supplementary Material

Supplementary figures, table 1 and table legends, and video legends.

Supplementary table 2.

Supplementary table 3.

Supplementary video 1.

Supplementary video 2.

## Figures and Tables

**Table 1:** Key resources

REAGENT or RESOURCE	SOURCE	IDENTIFIER
**Antibodies**		
Mouse anti-troponin T (cardiac/slow)	DSHB	Cat# CT3
Rabbit anti-NKX2.5	Santa Cruz	Cat# sc-14033
Mouse anti-alpha actinin (ACTN2)	Sigma	Cat# A7811
Rabbit anti-Phospho-AMPKα (Thr172)	Cell signaling	Cat# 2535
Rabbit anti- AMPKα	Cell signaling	Cat# 5831
Rabbit anti- Phospho-Akt (Ser473)	Cell signaling	Cat# 9271
Rabbit anti-AKT	Cell signaling	Cat# 9272
Rabbit anti-non-phospho (active) β-Catenin (Ser33/37/Thr41)	Cell signaling	Cat# 4270s
Rabbit anti-β-Catenin	Upstate	Cat# 06-734
Mouse Anti-phospho-Focal Adhesion Kinase (Tyr397)	Millipore	Cat# 05-1140
Mouse anti-β-actin	Santa Cruz	Cat# sc-47778
Peroxidase AffiniPure Goat Anti-Rabbit IgG (H+L)	Jackson ImmunoResearch	Cat# 111-035-144
Peroxidase AffiniPure Goat Anti-Mouse IgG (H+L)	Jackson ImmunoResearch	Cat# 115-035-146
Alexa Fluor® 594 AffiniPure™ Donkey Anti-Rabbit IgG (H+L)	Jackson ImmunoResearch	Cat# 711-585-152
Alexa Fluor® 488 AffiniPure Goat Anti-Mouse IgG	Jackson ImmunoResearch	Cat# 115-545-071
**Chemicals, Peptides, and Recombinant Proteins**		
DMEM/F12	Thermo Fisher Scientific	Cat# 11330-032
Penicillin/Streptomycin	Thermo Fisher Scientific	Cat# 15140-122
Holo-Transferrin human	Sigma-Aldrich	Cat# T0665
L-Ascorbic acid 2-phosphate sesquimagnesium salt hydrate	Sigma-Aldrich	Cat# A8960
Sodium Selenite	Sigma-Aldrich	Cat# S5261
Recombinant Human TGF-β1	Peprotech	Cat# 100-21
Recombinant Human FGF2	Chen lab, University of Macau	N/A
Insulin	Sigma-Aldrich	Cat# I9278
Matrigel	Corning	Cat# 354230
ROCK inhibitor Y-27632	DC chemical	Cat# DC1028
DPBS	Thermo Fisher Scientific	Cat# 14190-144
0.5M EDTA	Thermo Fisher Scientific	Cat# AM9262
Chemically Defined Lipid Concentrate	Thermo Fisher Scientific	Cat# 11905031
Alexa Fluor™ 555 Phalloidin	Thermo Fisher Scientific	Cat# A34055
Hoechst 33258	Thermo Fisher Scientific	Cat# H1398
IWP2	Selleck	Cat# S7085
CHIR-99021 HCl	Selleck	Cat# S2924
PP2	Enzo	Cat# BML-EI297
Y11	Tocris	Cat# 4498
TrypLE Select Enzyme (1X)	Thermo Fisher Scientific	Cat# 12563-029
Chemically Defined Lipid Concentrate	Thermo Fisher Scientific	Cat# 11905031
Bovine serum albumin (BSA)	Sigma	Cat# A7030
Gibco™ PSC Cardiomyocyte Maintenance Medium	Thermo Fisher Scientific	Cat# A2920801
**Commercial Assays**		
Click-iT® Plus EdU Flow Cytometry Assay Kit	Molecular Probes	Cat# C10633
RNAiso Plus reagent	Takara	Cat# 9109
High-Capacity cDNA Reverse Transcription Kit	Applied Biosystems	Cat# 4368814
TB Green Premix Ex Taq (Tli RNase H Plus)	Takara	Cat# RR420
Nextera XT DNA Library Preparation Kit	Illumina	Cat# FC-131-1024
SuperSignal™ West Pico PLUS Chemiluminescent Substrate	Thermo Fisher Scientific	Cat# 34577
SuperSignal™ West Dura Extended Duration substrate	Thermo Fisher Scientific	Cat# 34075
BCA protein assay kit	Thermo Fisher Scientific	Cat# RE232675
**Deposited data**		
RNA-seq data	This paper	GEO: GSE262203, GEO: GSE271094
scRNA-seq data	This paper	GEO: GSE262425
**Experimental Models: Cell Lines**		
H1 hESC line	WiCell Research Institute	NIHhESC-10-0043
H9 hESC line	WiCell Research Institute	NIHhESC-10-0062
NL-4 hiPSC line	National Institutes of Health (National Center for Regenerative Medicine)	NCRM-4
**Primers**		
Listed in Table S1	N/A	N/A
**Software and Algorithms**		
R version 4.1.2		https://www.r-project.org/
