# Supplementary material for: Remodeling Cell Adhesion Releases Cardiac Potential of Human Pluripotent Stem Cells with Continuous Proliferation and Accelerated Maturation
Source: Int J Biol Sci. 2025 Oct 10;21(15):6542–58. doi: 10.7150/ijbs.120853 (PMC12631065; doi:10.7150/ijbs.120853)
Supplement: Supplementary file 1 — Supplementary figures, table 1 and table legends, and video legends. [file ijbsv21p6542s1.pdf]

Remodeling Cell Adhesion Releases the Cardiac Potential of Human Pluripotent Stem Cells with Continuous Proliferation and Accelerated Maturation

Weiwei Liu, Chuyu Liu, Qian Wang, Chengwu Li, Jiaxian Wang, Ning-Yi Shao, Guokai Chen

List of supplemental materials

- Supplemental figures and legends
- Supplemental video
    - Supplemental video 1. Beating cardiomyocytes induced by passaging
    - Supplemental video 2. Beating cardiomyocytes induced by passaging and IWP2
- Table S1. Primers used in quantitative PCR
- Table S2. qPCR data used to generate heatmaps
- Table S3. List of differentially expressed genes (DEGs) from RNA-seq analysis

Figure S1

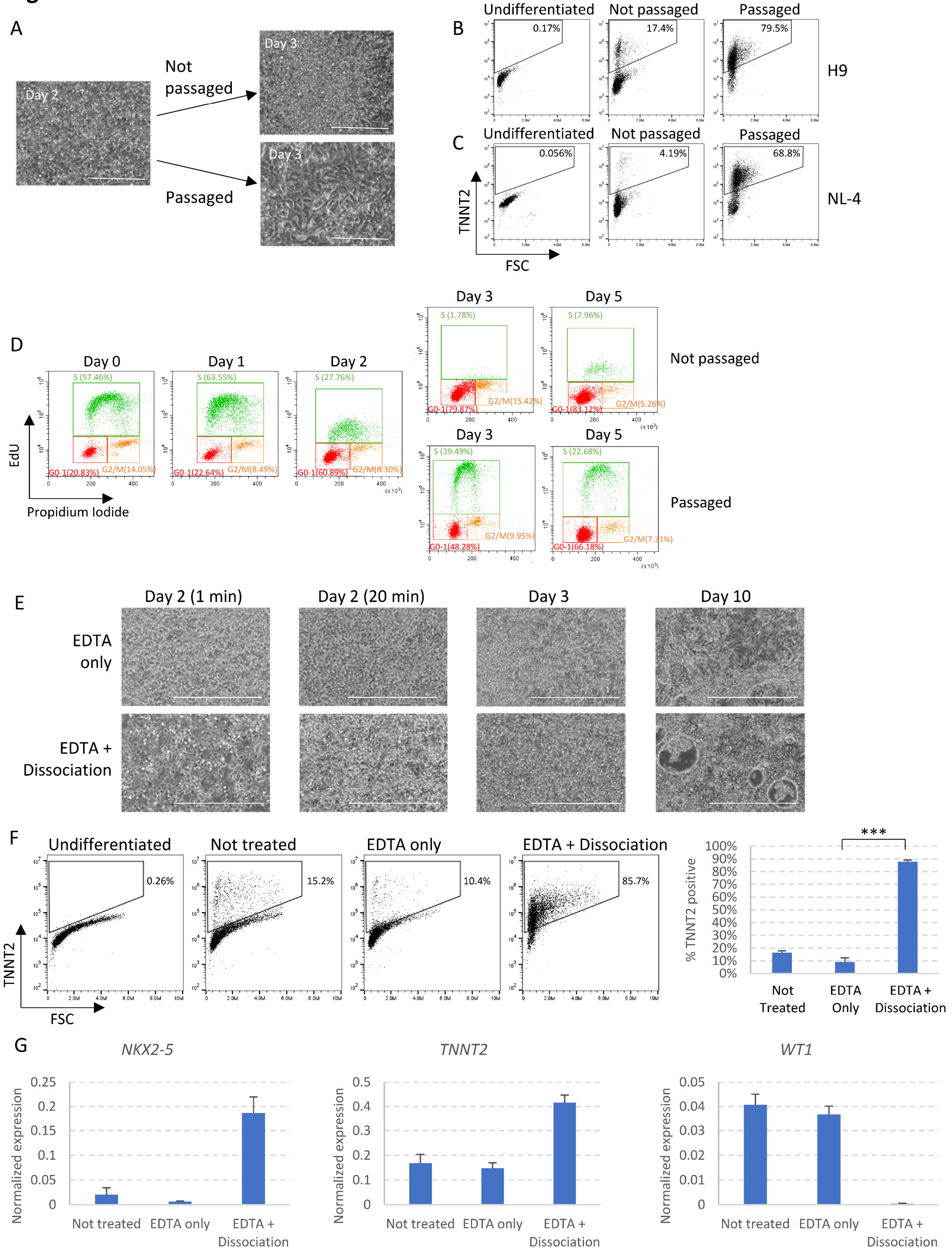

A
Day 2
Not passaged
Day 3
Passaged
Day 3
B
Undifferentiated
Not passaged
Passaged
0.17%
17.4%
79.5%
H9
C
Undifferentiated
Not passaged
Passaged
0.056%
4.19%
68.8%
NL-4
TNNT2
FSC
D
Day 0
Day 1
Day 2
Day 3
Day 5
S (57.46%)
G0-1(20.83%)
G2/M(14.05%)
S (63.55%)
G0-1(22.64%)
G2/M(8.49%)
S (27.76%)
G0-1(60.89%)
G2/M(8.30%)
S (1.78%)
G0-1(79.87%)
G2/M(15.42%)
S (7.96%)
G0-1(83.12%)
G2/M(5.26%)
Not passaged
S (39.49%)
G0-1(48.28%)
G2/M(9.95%)
S (22.68%)
G0-1(66.18%)
G2/M(7.31%)
Passaged
EdU
Propidium Iodide
E
Day 2 (1 min)
Day 2 (20 min)
Day 3
Day 10
EDTA only
EDTA + Dissociation
F
Undifferentiated
Not treated
EDTA only
EDTA + Dissociation
0.26%
15.2%
10.4%
85.7%
TNNT2
FSC
% TNNT2 positive
***
Not Treated
EDTA Only
EDTA + Dissociation
G
NKX2-5
TNNT2
WT1
Normalized expression
Not treated
EDTA only
EDTA + Dissociation

Figure S1. Dissociation of cell-matrix adhesion releases cardiac potential during critical period of cell fate determination. Related to Figure 1.

A) Phase contrast images of Day 3 cells. Scale bar, 400μm. B-C) Impact of Cell adhesion remodeling on spontaneous differentiation of mesendoderm progenitors induced from H9 hESC (B) and NL-4 iPSC (C) lines. $TNNT2^{+}$ cells were analyzed by flow cytometry on Day 10 of differentiation. D) Cell cycle analysis using Edu and propidium iodide (PI). Red, G0-1 phase; Orange, G2/M phase; Green, S phase. Data are representative of three independent experiments. E) Comparison of cells treated with EDTA and dissociated / replated on Day 2 (bottom row) versus those treated with EDTA but not dissociated on Day 2 (top row). Phase contrast images were taken right after treatment (1 min) on Day 2, 20 minutes after treatment on Day 2, and on Day 3 and Day 10 of differentiation. Scale bar, 400μm. F) Flow cytometry analysis of $TNNT2^{+}$ cells on Day 10 of differentiation, comparing untreated, EDTA-treated and EDTA-treated + dissociated cells. Data presented as mean ± SD of three biological replicates. ***, $p < 0.001$ (One-Way ANOVA with Dunnett's Multiple Comparison Test). G) qPCR analysis on Day 10 of differentiation, comparing untreated, EDTA-treated and EDTA-treated + dissociated cells. Data are normalized to *GAPDH* and presented as mean ± SD of three biological replicates.

Figure S2

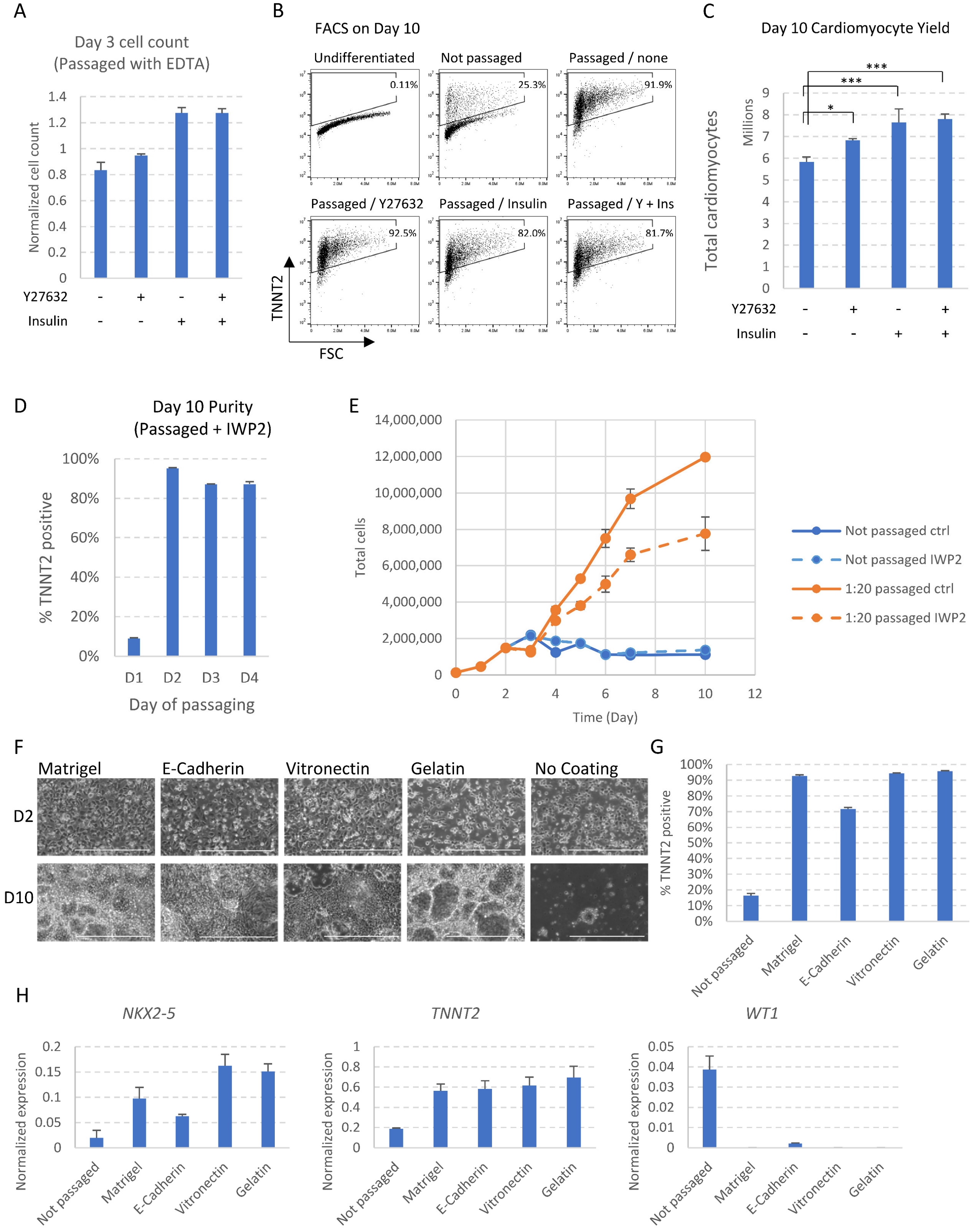
A
Day 3 cell count
(Passaged with EDTA)
Normalized cell count
Y27632
Insulin
B
FACS on Day 10
Undifferentiated
Not passaged
Passaged / none
Passaged / Y27632
Passaged / Insulin
Passaged / Y + Ins
0.11%
25.3%
91.9%
92.5%
82.0%
81.7%
TNNT2
FSC
C
Day 10 Cardiomyocyte Yield
Total cardiomyocytes
Millions
D
Day 10 Purity
(Passaged + IWP2)
% TNNT2 positive
D1
D2
D3
D4
Day of passaging
E
Total cells
Time (Day)
Not passaged ctrl
Not passaged IWP2
1:20 passaged ctrl
1:20 passaged IWP2
F
Matrigel
E-Cadherin
Vitronectin
Gelatin
No Coating
D2
D10
G
% TNNT2 positive
Not passaged
Matrigel
E-Cadherin
Vitronectin
Gelatin
H
NKX2-5
TNNT2
WT1
Normalized expression

Figure S2. Synergistic signaling modulation maximizes cardiomyocyte production after cell dissociation. Related to Figure 2.

A) Cell count on Day 3, 24 hours after cell dissociation and replating on Day 2 using EDTA method. Cell numbers were normalized to the number of cells seeded on Day 2. Data presented as mean ± SD of three biological replicates. B-C) FACS analysis of $TNNT2^+$ cells on Day 10 of differentiation, showing the impact of ROCK inhibitor Y27632 and insulin. Cells were induced toward cardiomyocytes by dissociation on Day 2 in the presence or absence of Y27632 (5uM) or insulin (1μg/ml), applied on Day 2. Data presented as mean ± SD of three biological replicates. *, $p < 0.05$; ***, $p < 0.001$ compared to control (One-Way ANOVA with Dunnett's Multiple Comparison Test). D) FACS analysis of $TNNT2^+$ cells on Day 10 of differentiation, comparing cells dissociated and replated on different days in the differentiation process with IWP2 applied Day 2-4. Data presented as mean ± SD of three biological replicates. E) Cell growth curve through the differentiation process. Cells were passaged on Day 2 at a split ratio of 1:20 and compared to non-passaged cells. IWP2 was applied Day 2-4. Data presented as mean ± SD of three biological replicates, and numbers represent the total cell count generated from one initial well on a 24-well plate. F) Morphology of cells plated in Matrigel-, E-Cadherin-, vitronectin-, gelatin-coated or uncoated plates after dissociation on Day 2. Phase contrast images were taken on Day 2 (2 hours after dissociation) and Day 10 of differentiation. Scale bar, 400μm. G) FACS analysis of $TNNT2^+$ cells on Day 10 of differentiation, comparing cells plated on different substrates. Data presented as mean ± SD of three biological replicates. H) qPCR analysis on Day 10 of differentiation, comparing cells plated on different substrates. Results are normalized to *GAPDH*. Data presented as mean ± SD of three biological replicates.

Figure S3

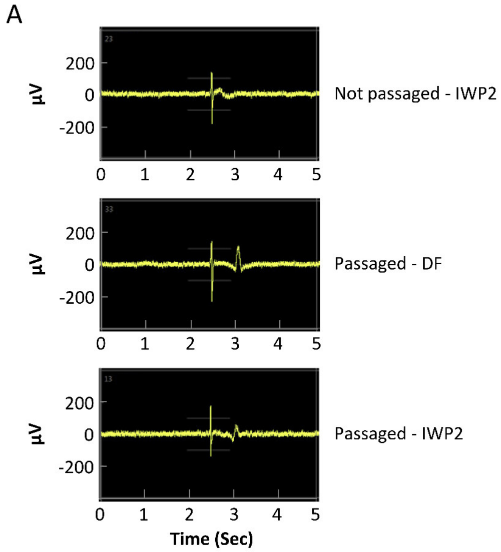

Figure S3. Characterization of passaging-induced cardiomyocytes. Related to Figure 3.

A) Representative electric activity recordings of cardiomyocytes induced by IWP2, passaging, and combined treatment with both IWP2 and passaging, measured on microelectrode array (MEA).

Figure S4

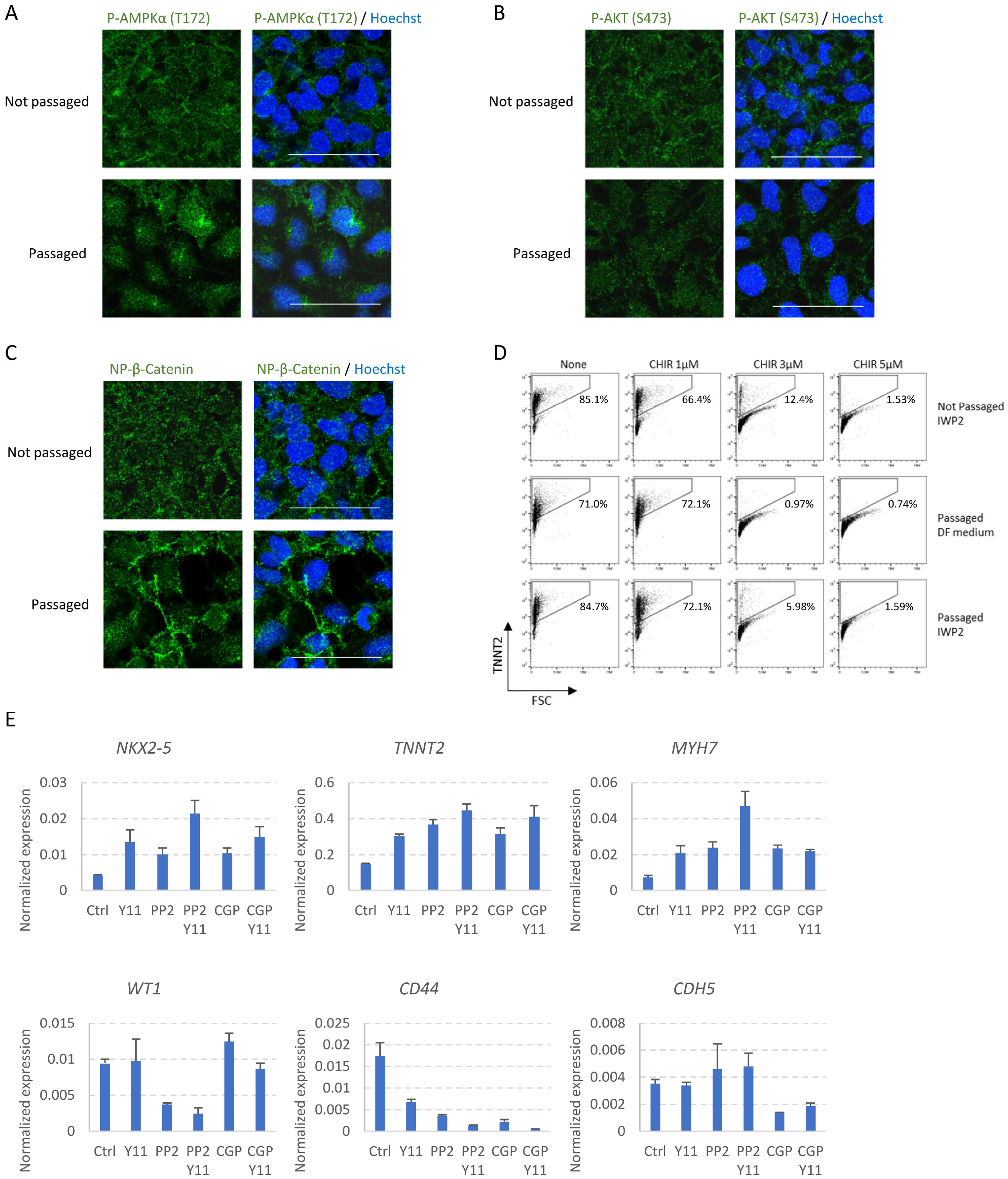
A
P-AMPKα (T172)
P-AMPKα (T172) / Hoechst
Not passaged
Passaged
B
P-AKT (S473)
P-AKT (S473) / Hoechst
Not passaged
Passaged
C
NP-β-Catenin
NP-β-Catenin / Hoechst
Not passaged
Passaged
D
None
CHIR 1μM
CHIR 3μM
CHIR 5μM
85.1%
66.4%
12.4%
1.53%
Not Passaged IWP2
71.0%
72.1%
0.97%
0.74%
Passaged DF medium
84.7%
72.1%
5.98%
1.59%
Passaged IWP2
TNNT2
FSC
E
NKX2-5
TNNT2
MYH7
WT1
CD44
CDH5
Normalized expression
Ctrl
Y11
PP2
PP2 Y11
CGP
CGP Y11

Figure S4. Cell adhesion remodeling regulates AMPK and AKT signaling through the integrin pathway. Related to Figure 4.

A-C) Immunostaining showing the impact of passaging on phosphorylated AMPKα (T172), phosphorylated AKT (S473), and non-phosphorylated (active) β-catenin. Mesendoderm progenitors (derived from H1 cells as shown in Figure 1A) on Day 2 of differentiation were either passaged using EDTA or changed to fresh DF medium without passaging, and fixed for immunostaining 1-2 hours after manipulation (1 hour for P-AMPK, 2 hours for P-AKT and NP-β-catenin). Nuclei were stained with Hoechst (blue). Scale bar, 50μm. D) Flow cytometry analysis of $TNNT2^{+}$ cells on Day 10, showing impact of CHIR99021 (applied Day 2-4) on cardiac differentiation induced by IWP2 (applied to non-passaged cells Day 2-4), passaging, or both. E) qPCR analysis on Day 10 of differentiation, showing the impact of SRC inhibitor (PP2, 5μM or CGP77675, 5uM) and/or FAK inhibitor (Y11, 10μM) on cell fate. Treatments were applied on Day 2 for 6 hours, followed by culturing in DF medium with daily medium changes. Data are normalized to *GAPDH* and presented as mean ± SD of three biological replicates.

Figure S5

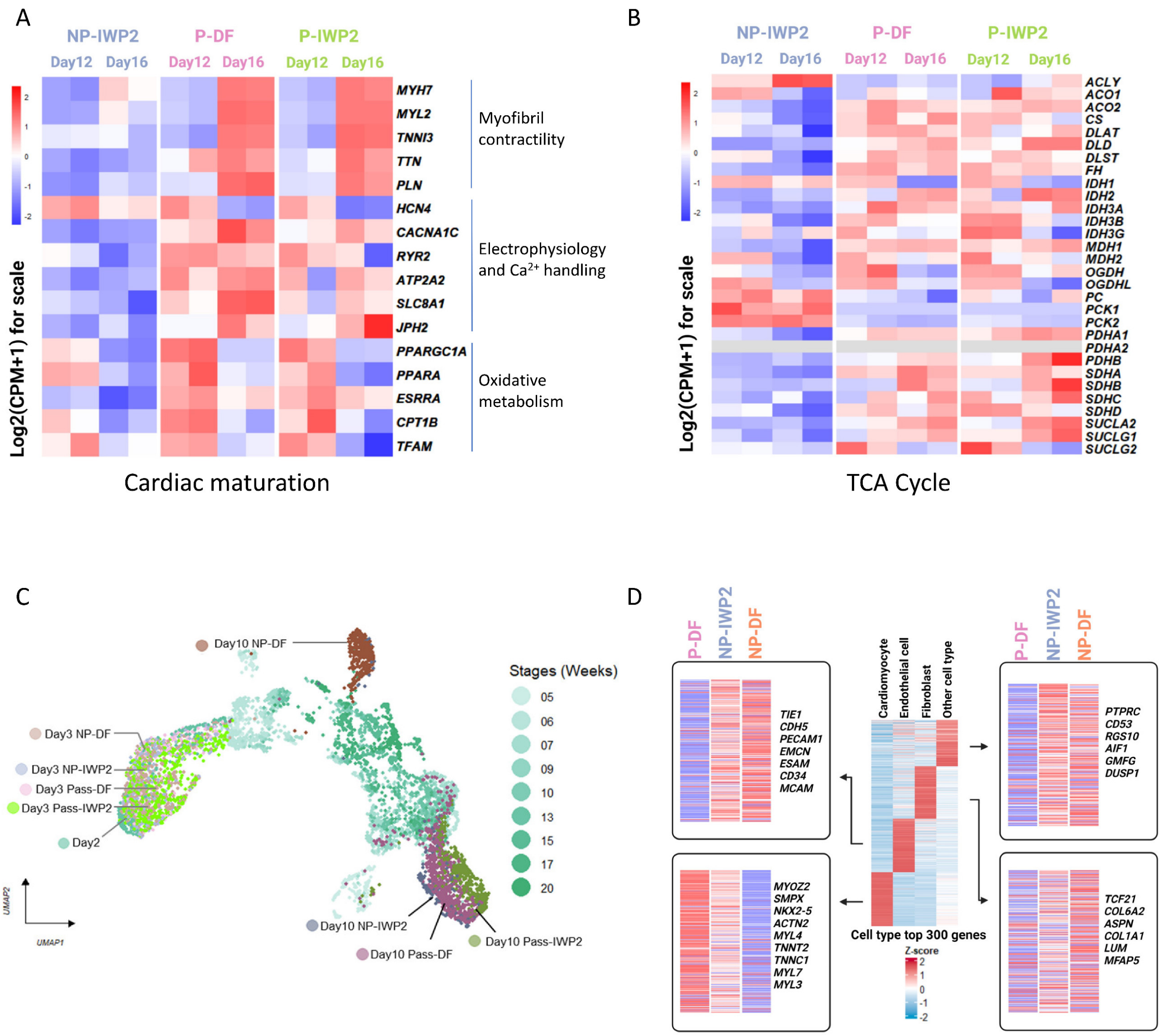
A
NP-IWP2
P-DF
P-IWP2
Day12
Day16
MYH7
MYL2
TNNI3
TTN
PLN
HCN4
CACNA1C
RYR2
ATP2A2
SLC8A1
JPH2
PPARGC1A
PPARA
ESRRA
CPT1B
TFAM
Myofibril contractility
Electrophysiology and Ca2+ handling
Oxidative metabolism
Log2(CPM+1) for scale
Cardiac maturation
B
ACLY
ACO1
ACO2
CS
DLAT
DLD
DLST
FH
IDH1
IDH2
IDH3A
IDH3B
IDH3G
MDH1
MDH2
OGDH
OGDHL
PC
PCK1
PCK2
PDHA1
PDHA2
PDHB
SDHA
SDHB
SDHC
SDHD
SUCLA2
SUCLG1
SUCLG2
TCA Cycle
C
Day10 NP-DF
Day3 NP-DF
Day3 NP-IWP2
Day3 Pass-DF
Day3 Pass-IWP2
Day2
Day10 NP-IWP2
Day10 Pass-DF
Day10 Pass-IWP2
Stages (Weeks)
05
06
07
09
10
13
15
17
20
UMAP1
UMAP2
D
P-DF
NP-IWP2
NP-DF
TIE1
CDH5
PECAM1
EMCN
ESAM
CD34
MCAM
MYOZ2
SMPX
NKX2-5
ACTN2
MYL4
TNNT2
TNNC1
MYL7
MYL3
Cardiomyocyte
Endothelial cell
Fibroblast
Other cell type
Cell type top 300 genes
Z-score
PTPRC
CD53
RGS10
AIF1
GMFG
DUSP1
TCF21
COL6A2
ASPN
COL1A1
LUM
MFAP5

Figure S5. Cell adhesion remodeling accelerates cardiac fate determination and maturation. Related to Figure 5.

A) Heatmap showing the expression of cardiac maturation marker genes on Day 12 and 16 in cardiomyocytes induced by IWP2 (NP-IWP2), passaging (P-DF), or both (P-IWP2), generated from bulk RNA sequencing data. B) Heatmap showing the expression of TCA cycle genes on Day 12 and 16, generated from bulk RNA sequencing data. C) Uniform Manifold Approximation and Projection (UMAP) plots visualization of human embryonic heart cells (GSE106118) (Cui et al., 2019) by developmental stage, integrated with scRNA-seq data of H1-derived cells. Different shades of green represent gestational weeks in human embryonic heart cell data. D) Middle: Heatmap displaying the Z-score-scaled average expression levels of the top 300 DEGs in human embryonic heart cell types. Left and right: Heatmaps showing the expression of top 300 DEGs from human embryonic heart cells in H1-derived cells generated under different treatments, based on Day 10 bulk RNA-seq results.

Supplemental Video 1. Beating cardiomyocytes induced by passaging. Related to Figure 1. Mesendoderm progenitors were passaged on Day 2 of differentiation, and then cultured in DF medium (DMEM/F12, ascorbic acid, selenite, transferrin, chemically defined lipid, penicillin/streptomycin) without other inducers. Video taken on Day 8.

Supplemental Video 2. Beating cardiomyocytes induced by passaging combined with IWP2. Related to Figure 2. Mesendoderm progenitors were passaged on Day 2 of differentiation, treated with IWP2 (applied Day 2-4 in DF medium), and then cultured in DF medium. Video taken on Day 8.

Table S1. Sequence of primers used for quantitative PCR

Gene	Forward primer	Reverse primer
NKX2-5	CAAGTGTGCGTCTGCCTTT	CAGCTCTTTCTTTTCGGCTCTA
TNNT2	GGAGGAGTCCAAACCAAAGCC	TCAAAGTCCACTCTCTCTCCATC
MYH7	ACTGCCGAGACCGAGTATG	GCGATCCTTGAGGTTGTAGAGC
MYL7	ACATCATCACCCATGGAGACGAGA	GCAACAGAGTTTATTGAGGTGCCC
MYL2	TGTCCCTACCTTGTCTGTTAGCCA	ATTGGAACATGGCCTCTGGATGGA
WT1	ATAGGCCAGGGCATGTGTATGTGT	AGTTGCCTGGCAGAACTACATCCT
CD44	TGGCACCCGCTATGTCGAG	GTAGCAGGGATTCTGTCTG
CD73	CTCCTCTCAATCATGCCGCT	CATCAATGGGCGACCGGATA
PECAM	TCTATGACCTCGCCCTCCACAAA	GAACGGTGTCTTCAGGTTGGTATTTCA
CDH5	TGGAGAAGTGGCATCAGTCAACAG	TCTACAATCCCTTGCAGTGTGAG
AFP	CAAACTATTGGCCTGTGGCG	TGGCCAACACCAGGGTTTAC
CDX2	GGAACCTGTGCGAGTGGAT	TCCGTGTACACCACTCGATATT
NANOG	TGTGATTTGTGGGCCTGAAG	AAGTGGGTTGTTTGCCTTTGG
POU5F1	CTTGAATCCCGAATGGAAAGGG	GTGTATATCCCAGGGTGATCCTC

Table S2. qPCR data used to generate heatmaps. Related to Figure 1B. This table shows results of qPCR analysis on Day 10 of differentiation, comparing cells differentiated in static culture (not passaged), cells passaged 1:6 on Day 2, and undifferentiated H1 cells.

Table S3. List of differentially expressed genes (DEGs) from RNA-seq analysis. Related to Figure 3A. This table shows the expression levels of DEGs corresponding to each cluster in the heatmap in Figure 3A.
